# EETs/sEHi alleviates nociception by blocking the crosslink between endoplasmic reticulum stress and neuroinflammation in a central poststroke pain model

**DOI:** 10.1186/s12974-021-02255-3

**Published:** 2021-09-16

**Authors:** Tongtong Liu, Ting Li, Xuhui Chen, Zuofan Li, Miaomiao Feng, Wenlong Yao, Li Wan, Chuanhan Zhang, Yue Zhang

**Affiliations:** 1 Department of Anesthesiology, Tongji Hospital, Tongji Medical College, Huazhong University of Science and Technology, Wuhan, 430030 Hubei Province People’s Republic of China; 2 Department of Ophthalmology, Tongji Hospital, Tongji Medical College, Huazhong University of Science and Technology, Wuhan, 430030 Hubei Province People’s Republic of China

**Keywords:** Central post-stroke pain, Soluble epoxide hydrolase, Endoplasmic reticulum stress, Neuroinflammation, MAPK signaling

## Abstract

**Background:**

Central post-stroke pain (CPSP) is a chronic and intolerable neuropathic pain syndrome following a cerebral vascular insult, which negatively impacts the quality of life of stroke survivors but currently lacks efficacious treatments. Though its underlying mechanism remains unclear, clinical features of hyperalgesia and allodynia indicate central sensitization due to excessive neuroinflammation. Recently, the crosslink between neuroinflammation and endoplasmic reticulum (ER) stress has been identified in diverse types of diseases. Nevertheless, whether this interaction contributes to pain development remains unanswered. Epoxyeicosatrienoic acids (EETs)/soluble epoxy hydrolase inhibitors (sEHi) are emerging targets that play a significant role in pain and neuroinflammatory regulation. Moreover, recent studies have revealed that EETs are effective in attenuating ER stress. In this study, we hypothesized that ER stress around the stroke site may activate glial cells and lead to further inflammatory cascades, which constitute a positive feedback loop resulting in central sensitization and CPSP. Additionally, we tested whether EETs/sEHi could attenuate CPSP by suppressing ER stress and neuroinflammation, as well as their vicious cycle, in a rat model of CPSP.

**Methods:**

Young male SD rats were used to induce CPSP using a model of thalamic hemorrhage and were then treated with TPPU (sEHi) alone or in combination with 14,15-EET or 14,15-epoxyeicosa-5(Z)-enoic acid (14,15-EEZE, the EET antagonist), tunicamycin (Tm, ER stress inducer), or 4-PBA (ER stress inhibitor). Nociceptive behaviors, ER stress markers, JNK and p38 (two well-recognized inflammatory kinases of mitogen-activated protein kinase (MAPK) signaling) expression, and glial cell activation were assessed. In addition, some healthy rats were intrathalamically microinjected with Tm or lipopolysaccharide (LPS) to test the interaction between ER stress and neuroinflammation in central pain.

**Results:**

Analysis of the perithalamic lesion tissue from the brain of CPSP rats demonstrated decreased soluble epoxy hydrolase (sEH) expression, which was accompanied by increased expression of ER stress markers, including BIP, p-IRE, p-PERK, and ATF6. In addition, inflammatory kinases (p-p38 and p-JNK) were upregulated and glial cells were activated. Intrathalamic injection of sEHi (TPPU) increased the paw withdrawal mechanical threshold (PWMT), reduced hallmarks of ER stress and MAPK signaling, and restrained the activation of microglia and astrocytes around the lesion site. However, the analgesic effect of TPPU was completely abolished by 14,15-EEZE. Moreover, microinjection of Tm into the thalamic ventral posterior lateral (VPL) nucleus of healthy rats induced mechanical allodynia and activated MAPK-mediated neuroinflammatory signaling; lipopolysaccharide (LPS) administration led to activation of ER stress along the injected site in healthy rats.

**Conclusions:**

The present study provides evidence that the interaction between ER stress and neuroinflammation is involved in the mechanism of CPSP. Combined with the previously reported EET/sEHi effects on antinociception and neuroprotection, therapy with agents that target EET signaling may serve as a multi-functional approach in central neuropathic pain by attenuating ER stress, excessive neuroinflammation, and subsequent central sensitization. The use of these agents within a proper time window could not only curtail further nerve injury but also produce an analgesic effect.

## Background

Central post-stroke pain (CPSP) is a neuropathic pain syndrome that arises after lesions or diseases affecting the central nervous system due to cerebrovascular events [1]. This syndrome is characterized by pain and sensory disorders in body parts that correspond to the damaged brain region due to cerebrovascular accidents [2]. This intolerable and prolonged painful condition after stroke reduces patients’ quality of life, induces mood disorders and sleep disturbances, and decreases social ability [2, 3]. Although the reported prevalence of CPSP ranges from 8 to 35% [4], considering the aging trend of the population and the higher susceptibility of stroke in the elderly, CPSP is a far underestimated and underreported phenomenon. Diverse approaches, including pharmacological and non-pharmacological regimens, have been used to treat this disease; however, their therapeutic efficacy is far from satisfactory and often results in adverse side effects [1, 3]. Consequently, elucidating the mechanisms underlying the pathologies of CPSP is important.

The pathological state of stroke (ischemia, hypoxia, and hypoglycemia) can fluctuate the well-orchestrated process of protein folding, which leads to the accumulation of unfolded proteins in the endoplasmic reticulum (ER) and triggers a condition termed ER stress [5]. In this context, the unfolded protein response (UPR), which is an evolutionarily conserved mechanism in eukaryotic cells, is activated to reestablish ER homeostasis [6, 7]. During this process, three canonical transmembrane sensors, including inositol requiring enzyme1α (IRE1α), protein kinase RNA-like ER kinase (PERK), and activating transcription factor 6 (ATF6), are engaged in the initiation of UPR [6, 8]. These sensors are usually inactive by binding their intraluminal domains to the ER chaperone binding immunoglobulin protein (BIP) under unstressed conditions. However, intense or prolonged ER stress can lead to chronic inflammation, apoptosis, and autophagy [9–11].

Accumulating evidence indicate that partial or full-scale activation of ER stress in the peripheral nervous system and spinal dorsal horn plays an essential role in inflammatory and neuropathic pain [12–15]. More recently, ER stress has been linked to synaptic transmission and neuroexcitation, and a report by Nosyreva et al. revealed that neurons treated with an ER stress inducer, tunicamycin (Tm), exhibited increased sensitivity of spontaneous release machinery to calcium (Ca^2+^), which resulted in augmentation of excitatory spontaneous neurotransmission [16]. In an in vitro model of cerebral ischemia, Maier et al. found that in neurons, ER stress-mediated binding of the transcription factor C/EBP homologous protein (CHOP) prevented heterodimerization of gamma-aminobutyric acid B receptors (GABA_B_R) and their subsequent trafficking to the cell surface, which diminished GABA_B_ signaling and thus neuronal inhibition [17]. Taken together, this augmentation in excitatory neurotransmission and neuronal disinhibition induced by ER stress leads to central sensitization, which may contribute to the explanation of central pain after stroke.

Prolonged UPR is also an inflammatory nidus that may elicit a defensive innate immune response against invading pathogens. In the brain, neuroinflammation is characterized by astrocyte and microglial cell activation, consequently leading to the release of cytokines and chemokines such as interleukin-1 β (IL-1β), interleukin-6 (IL-6), and tumor necrosis factor-α (TNF-α) [18, 19]. The released pro-inflammatory agents further activate glial cells and lead to a hyper-release of cytokines from the trauma site. The sustained overload of protein processing during the neuroinflammation cascade leads to excessive ER stress, which constitutes a positive feedback loop resulting in central sensitization. Approaches targeting these harmful interactions between ER stress and neuroinflammation may provide a therapeutic option for CPSP.

Epoxyeicosatrienoic acids (EETs), the cytochrome P450 (CYP) metabolites of arachidonic acid (ARA), are widely distributed in the brain and are shown to be important modulators of cerebral blood flow regulation, axonal growth, and neuronal survival. Beyond these effects, EETs have attracted considerable attention because of their potential to modulate nociception [20, 21]. Endogenous EETs are rapidly degraded to their corresponding inactive diols or dihydro-eicosatrienoic acids (DHETs) within seconds by soluble epoxide hydrolase (sEH) (encoded by EPHX2) [22, 23]. This short in vivo half-life restrained EET application in clinical practice [21]. However, the development of sEH inhibitors (sEHi) can stabilize EETs levels and thereby prolong their half-life, which represents a possible strategy for improving the biological activity of EETs [21]. Our previous work revealed that EETs/sEHi influenced central sensitization and subsequently alleviated pain behavior after thalamic hemorrhagic stroke [24]. Moreover, mounting evidence has revealed that EETs can exert anti-inflammatory effects by inhibiting the expression of vascular adhesion molecules, NF-κB-mediated inflammatory cytokine secretion, and COX-2 gene induction [20, 25]. In addition, Ren et al. recently reported that sEHi protected against 1-methyl-4-phenyl-1,2,3,6-tetrahydropyridine (MPTP)-induced ER stress in the brain [26]. Based on these observations, we investigated whether the analgesic effects of EETs/sEHi are involved in curtailing the crosslink between ER stress and neuroinflammation in the brain. In the present study, we investigated the interactions among EETs/sEHi, ER stress, and neuroinflammation in the development of CPSP in a rat model of thalamic hemorrhagic stroke.

## Materials and methods

### Experimental animals

Young male Sprague-Dawley rats (280–300 g, 10–12 weeks old, Vital River Experimental Animal Corporation of Beijing, China) were used in this study. Animals were housed in an exhaust ventilated closed-system cage with free access to food and water at Tongji Hospital Experimental Animal Research Center (Wuhan, Hubei, China) under standard conditions (temperature: 22−25 °C, humidity: 60% ± 15%, 12-h alternate light-dark cycles, 2 per cage). To exclude the confounding factors induced by the circadian rhythm and environmental changes, all animals were acclimated for 1 week before the experiments, and all animal experiments were performed between 9.00 am and 6.00 pm. The experimental animal protocols were reviewed and approved by the Animal Care and Use Committee of Tongji Hospital, Tongji Medical College, Huazhong University of Science and Technology, Wuhan, China. The research was performed according to the guidelines of the Committee for Research and Ethical Issues of the International Association for the Study of Pain and in strict accordance with the National Institutes of Health Guide for the Care and Use of Laboratory Animals (NIH Publications No. 8023, revised 1978).

### Chemicals

Type IV collagenase (C5138, Sigma-Aldrich, St. Louis, MO) was dissolved in 0.9% saline and injected at a dose of 0.025 U/0.25 μl per rat. 1-Trifluoromethoxyphe-nyl-3-(1-propionylpiperidin-4-yl)urea (TPPU, a selective sEHi) was purchased from Cayman Chemical, initially dissolved in 0.1% dimethyl sulfoxide (DMSO) as a stock solution, and diluted to 0.01, 0.1, and 1 mM as the working solution before each study. As EETs are highly regio-specifically metabolized by sEH, and 14,15-EET is the preferred substrate of sEH to convert to the less active 14,15-DHET [27], exogenous 14,15-EET was chosen for co-application with TPPU in some groups of CPSP rats in this study. Authentic 14,15-EET (0.1 μg per rat) and 14, 15-EET antagonist 14,15-epoxyeicosa-5(Z)-enoic acid (14,15-EEZE, 3.25 ng per rat) were purchased from Cayman Chemical (Ann Arbor, MI), initially dissolved in 0.1% DMSO as stock solution, and diluted with 0.1% dimethyl sulfoxide (DMSO). Tunicamycin (Tm), an activator of ER stress, was obtained from Sigma, dissolved in 0.1% DMSO, and used at doses of 0.01, 0.1, and 1 μg for intrathalamic injection. Lipopolysaccharides (LPS) from *Escherichia coli* O111:B4 (product no. L2630, Sigma) and the ER stress inhibitor 4-phenylbutyrate (4-PBA product no. SML0309, Sigma) were dissolved in sterile 0.9% saline at a concentration of 1 mg/μl, and used at a dose of 1 μg per rat for intrathalamic injection. The vehicles used in the present study were determined using the corresponding solvent medium, which was usually 0.1% DMSO or 0.9% saline.

### Experimental design

Blinding was done to control for factors that would affect the results of the experiments. The rats were assigned to different groups using a random digital table generated by a computer, and the researchers who undertook the work of inducing the model as well as performing the drug administration, behavioral tests, Western blotting, immunostaining, ELISA, and data analysis were blinded to the group assignments. The syringes containing the vehicle or target drugs, which were randomly coded, were prepared by different persons. As males were more vulnerable to CPSP after thalamic stroke than females in clinical researc h[28], only male rats were used in the experiment.

### Preparation of the CPSP animal model

The details of the establishment of the CPSP model are described in our previous work [24]. In brief, rats were anesthetized with isoflurane (4% for induction and 2% for maintenance), and a vertical scalp incision was made after the caput was shaved and disinfected. Then, rats were stereotactically microinjected with type IV collagenase (0.025 U/0.25 μl) (CPSP group) or sterile saline (sham control group) into the ventral posterior lateral nucleus (VPL) of the thalamus with a 0.5-μl Hamilton syringe (Bonaduz, GR, Switzerland). The stereotaxic coordinates of 3.48 mm anteroposterior to bregma, 3.4 mm lateral to the midline, and 6.2 mm ventral to the skull surface on the right side were used for VPL microinjection [24].

### Intrathalamic cannula implantation and drug administration

An infusion guide cannula (internal diameter 0.38 mm, RWD Life Science, Shenzhen, China) was implanted 4.2 mm above the VPL on the right side of the caput. To test the involvement of ER stress and neuroinflammation in central pain, we microinjected Tm or LPS into the VPL region of healthy rats using a polyethylene catheter (PE-10) connected to a microsyringe needle (internal diameter, 0.20 mm; Gaoge Industrial and Trading, Shanghai, China) via a guide cannula (experiment 2). To test the analgesic effect of sEHi on CPSP, groups of CPSP rats were treated with vehicle or TPPU (in a concentration gradient from 0.01 to 1 mM) once daily within the first 5 days after CPSP induction (experiment 3). Since early treatment with TPPU alone exhibited short-term analgesia after CPSP induction, groups of CPSP rats were next treated with TPPU (1 mM/1 μl) in combination with exogenous 14,15-EET (0.1 μg) or the EET antagonist 14,15-EEZE (3.25 ng) within the first 5 days after CPSP induction, and then received repeat doses on days 12, 13, and 14 (experiment 4). To test the role of ER stress in sEHi-mediated analgesia, TPPU (1 mM/1 μl) was used in combination with Tm (0.1 μg) or 4-PBA (1 μg) for thalamic microinjection (experiments 5 and 6). Subsequently, all rats were processed for the von Frey test, which will be described later. Detailed experimental plans on animal assignment and medication are presented in each corresponding timeline diagram.

### Behavioral tests

Mechanical allodynia was evaluated using the von Frey test. In brief, a test kit containing applications of distinct filaments ranging from 2 to 26 g (2, 4, 6, 8, 10, 15, and 26 g) was gradually performed in an increasing manner, and the force was sustained until buckling occurred for approximately 5 s. The minimal force that elicits positive responses (licking, biting, and abrupt withdrawal of the hind paw) was recorded. Paw withdrawal mechanical threshold (PWMT) was defined as the average of three minimal forces taken in sequential trials, each separated by 5 min [24]. All rats were acclimated to the environment for 7 days before basal measurements were taken, as well as were also acclimated for 30 min before each test. To avoid circadian variations, we performed all behavioral tests in the morning (9 am to 12 am).

### Western blotting analysis

CPSP rats subjected to each treatment described previously were sacrificed under deep anesthesia with 10% isoflurane. The tissues from the perilesion site were homogenized in an ice-cold mixture of radioimmunoprecipitation assay (RIPA) lysis buffer (10 μl/mg for tissue, Boster Biological Technology, China) and phenylmethylsulfonyl fluoride (PMSF, Roche). Total proteins were collected by centrifugation at 12,000 × g for 30 min at 4 °C. Protein concentrations were determined and normalized using a BCA protein assay kit (Thermo Fisher Scientific, Waltham, MA, USA). The samples were then boiled at 95 °C in sodium dodecyl sulfate sample buffer for 15 min and stored at – 80 °C until use. Proteins (20–40 μg) were loaded to sodium dodecyl sulfate (SDS)-polyacrylamide gel electrophoresis. Subsequently, the proteins were transferred onto polyvinylidene fluoride membranes. After blocking with 5% bovine serum albumin (BSA) or non-fat dried milk in Tris-buffered saline and Tween 20 (TBST) for 1.5 h at room temperature, the membranes were incubated with specific primary antibodies (Table 1) overnight at 4 °C with gentle agitation, followed by incubation with horseradish peroxidase-labeled immunoglobulin G (Table 1). The blots were detected using an enhanced chemiluminescence kit (Thermo Scientific) and exposed to a ChemiDoc XRS imaging system (Bio-Rad, CA). Signals were detected and quantified using Quantity One software (Bio-Rad, CA, USA). If required, the combined horseradish peroxidase-labeled antibodies bound to membranes were removed using a commercial stripping solution (Applygen Technologies Inc.), and then the membranes were re-immunoblotted with the respective primary antibodies.

**Table 1:** Primary and secondary antibodies for Western blot and immunofluorescence staining

Antibody	Provider	Host	Catalog number	Dilution for WB	Dilution for IF
BIP	Affinity	Rabbit	AF5366	1:1000	1:150
p-IRE1	Affinity	Rabbit	AF7150	1:500	1:150
p-PERK	Affinity	Rabbit	DF7576	1:500	1:150
ATF6	Affinity	Rabbit	DF6009	1:1000	1:150
XBP1	Proteintech	Rabbit	25997-1-AP	1:1000	
p-eIF2a	Affinity	Rabbit	AF3087	1:500	
eIF2a	Proteintech	Rabbit	11170-1-AP	1:1000	
IRE	Proteintech	Rabbit	27528-1-AP	1:1000	
PERK	Proteintech	Rabbit	20582-1-AP	1:1000	
p-JNK	CST	Rabbit	#4671	1:500	
JNK	CST	Rabbit	#9251	1:500	
p-p38	CST	Rabbit	#9216	1:500	
p38	CST	Rabbit	#14451	1:500	
GAPDH	Proteintech	Mouse	60004-1-Ig	1:1000	
Tublin	Proteintech	Mouse	66031-1-Ig	1:1000	
NeuN	Abcam	Mouse	Ab104224		1:200
IBA1	Abcam	Goat	Ab5076		1:200
GFAP	Abcam	Mouse	MAB360		1:200
Anti-rabbit IgG HRP	Aspen	Goat	AS1107	1:4000	
Anti-mouse IgG HRP	Aspen	Goat	AS1106	1:4000	
Alexa Fluor 488-AffiniPure anti-mouse IgG	Jackson	Goat	115-545-003		1:500
Alexa Fluor 594-AffiniPure anti-rabbit IgG	Jackson	Goat	111-585-003		1:500
Alexa Fluor 488-AffiniPure anti-goat IgG	Jackson	Donkey	705-545-003		1:500
Alexa Fluor 594-AffiniPure anti-rabbit	Jackson	Donkey	711-585-152		1:500

Abbreviations: *BIP*, binding immunoglobulin protein; *IRE1*, inositol-requiring kinase 1; *PERK*, protein kinase RNA-like endoplasmic reticulum kinase; *ATF6*, activating transcription factor 6; *XBP1*, X-box-binding protein 1; *eIF2α*, eukaryotic initiation factor 2α; *JNK*, c-jun NH2-terminal kinase; *IBA1*, ionized calcium-binding adapter molecule 1; *NeuN*, neuronal nuclei; *GFAP*, glial fibrillary acidic protein; HRP, horseradish peroxidase

### Immunostaining

Rats exposed to each treatment were sacrificed under deep anesthesia and transcardially perfused with ice-cold 0.01 M phosphate-buffered saline (PBS) followed by 4% (m/v) paraformaldehyde. The brains were dissected and post-fixed with paraformaldehyde at 4 °C overnight and then dehydrated in 20% and 30% sucrose solution for 24 to 48 h at 4 °C. Then, the harvested brains were sectioned using a frozen microtome (thickness 15–20 μm). The sections were then permeabilized with 0.3% Triton X-100 (Beyotime Institute of Biotechnology, China) for 10 min and blocked with 10% goat serum (Boster, China) or 10–20% donkey serum (Abbkine, China) for 1.5 h at room temperature. Next, the sections were incubated with the primary antibodies (Table 1) overnight, followed by incubation with the appropriate secondary antibodies (Table 1). After washing with PBS, the sections were incubated with 4′,6-diamidino-2-phenylindole (DAPI, Abcam) for nucleic acid staining. Images were captured using a laser-scanning confocal microscope (Nikon, Tokyo, Japan). Quantification of immunoreactivity was accomplished by calculating the mean fluorescence intensity and the number of GFAP^+^ and IBA1^+^ cells of astrocytes and microglia using Image J (National Institutes of Health, Bethesda, MD, USA).

### ELISA

The tissues from the perilesion site were lysed in RIPA lysis buffer (10 μl/mg for tissue, Boster Biological Technology, China). TNF-α, IL-1β, and IL-6 concentrations in the cell lysate were measured with ELISA kits (E-EL-R2856c; E-EL-R0012c; E-EL-R0015c, Elabscience Biotechnology Co., Ltd., Wuhan, China) according to the manufacturer’s instructions, and the levels were detected with a microplate reader at a wavelength of 450 nm.

### Electron microscopic examination

Rats were deeply anesthetized with 10% isoflurane and perfused with PBS and 2.5% glutaraldehyde. The rat brains were then removed and kept in 2.5% glutaraldehyde at 4 °C. After being fixed in 1% osmium tetroxide and dehydrated in graded ethanol and embedded in epoxy resin, the samples were cut into 60-μm sections and stained with uranyl acetate and lead citrate. The images were captured using a Hitachi TEM system.

### Statistical analysis

Data are presented as mean ± standard deviation (SD). Data from the behavioral tests were analyzed using a two-way repeated measures analysis of variance (ANOVA) followed by post hoc tests (Bonferroni test) to assess differences at each time point between groups. Results from Western blotting were analyzed using a one-way analysis of variance (ANOVA) followed by the Bonferroni test or independent *t*-tests for between-group comparisons. Results from ELISA were analyzed using a one-way analysis of variance (ANOVA) followed by the Bonferroni test. Normal distribution was confirmed using the Shapiro-Wilk test and sphericity analysis. All comparisons were performed using GraphPad Prism 6.0 (GraphPad Software, San Diego, CA). All tests were two-tailed, and a value of *P* < 0.05 was considered statistically significant.

## Results

### CPSP rats exhibited mechanical allodynia and enhanced glia activation in the perithalamic lesion site

**Fig. 1:**
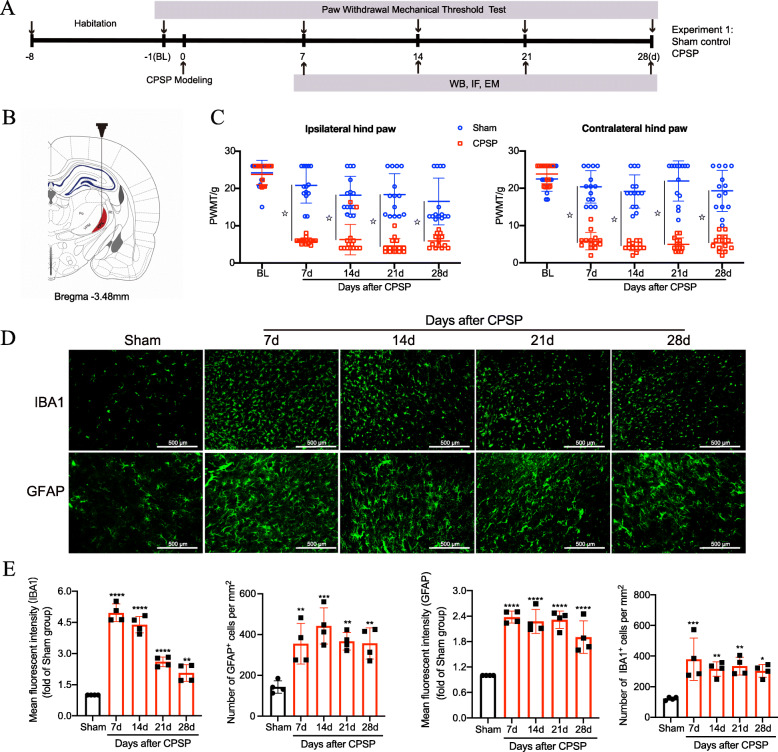
CPSP rats exhibited mechanical allodynia and glial cell activation. **a** The experimental timeline of surgical procedure, pain behavior tests, Western blot, immunostaining, and EM observation. **b** The schematic illustration of the stereotaxic intrathalamus microinjection of IV collagenase for the CPSP model. **c** CPSP rats exhibit a significant decrease in the PWMT in both ipsilateral and contralateral hind paws throughout 4 weeks after lesion compared with the sham control at each time point (*n* = 14 per group), two-way ANOVA, followed by Bonferroni tests, ^☆^*P* < 0.0001 versus the sham control. **d** Representative images of immunofluorescence staining with GFAP and IBA1 along the thalamic lesion site of CPSP rats (*n* = 4 rats per group). **e** Quantification of the mean fluorescent intensity and number of GFAP^+^ and IBA1^+^ cells along the perilesion site of CPSP rats. (*n* = 4 rats per group, *P < 0.05, **P < 0.01, ***P < 0.001, ****P < 0.0001, compared with the sham group. Scale bar = 500 μm). Changes in the morphology and number of microglia and astrocytes along the lesion site indicate activation of neuroinflammation under CPSP conditions. WB, western blotting; IF, immunofluorescence; EM, electron microscopic; BL, baseline

We induced a CPSP rat model by injecting collagenase into the VPL nucleus of the right thalamus. The timeline of surgery, behavioral tests, Western blot analysis, and immunostaining for experiment 1 are shown in Fig. 1a. The model scheme and hemorrhagic lesions of the rats are shown in Fig. 1b. Our previous work showed no significant difference between the CPSP rats and their sham control in terms of motor function and thermal threshold during the perioperative period of VPL hemorrhage, based on rotarod and plantar tests [24]. Consequently, we focused on mechanical allodynia in the present study. Compared with the sham control, CPSP rats exhibited decreased PWMT in both hind paws in response to von Frey brush stimulation beginning at day 7 after CPSP, which persisted for at least 28 days (Fig. 1c), indicating that thalamic hemorrhage produced significant mechanical allodynia at the 4-week observation period after the lesion. Meanwhile, the results of the quantitative analysis of immunostaining showed that compared with the sham group, the mean fluorescent intensity and positive cell numbers of GFAP and IBA1 in the perilesion site were markedly elevated at 7, 14, 21, and 28 days after hemorrhagic stroke (Fig. 1 d and e). This prolonged activation of glial cells (lasting at least 1 month) may help maintain mechanical allodynia in CPSP rats.

### CPSP rats exhibited increased sEH levels in the perithalamic lesion site

**Fig. 2:**
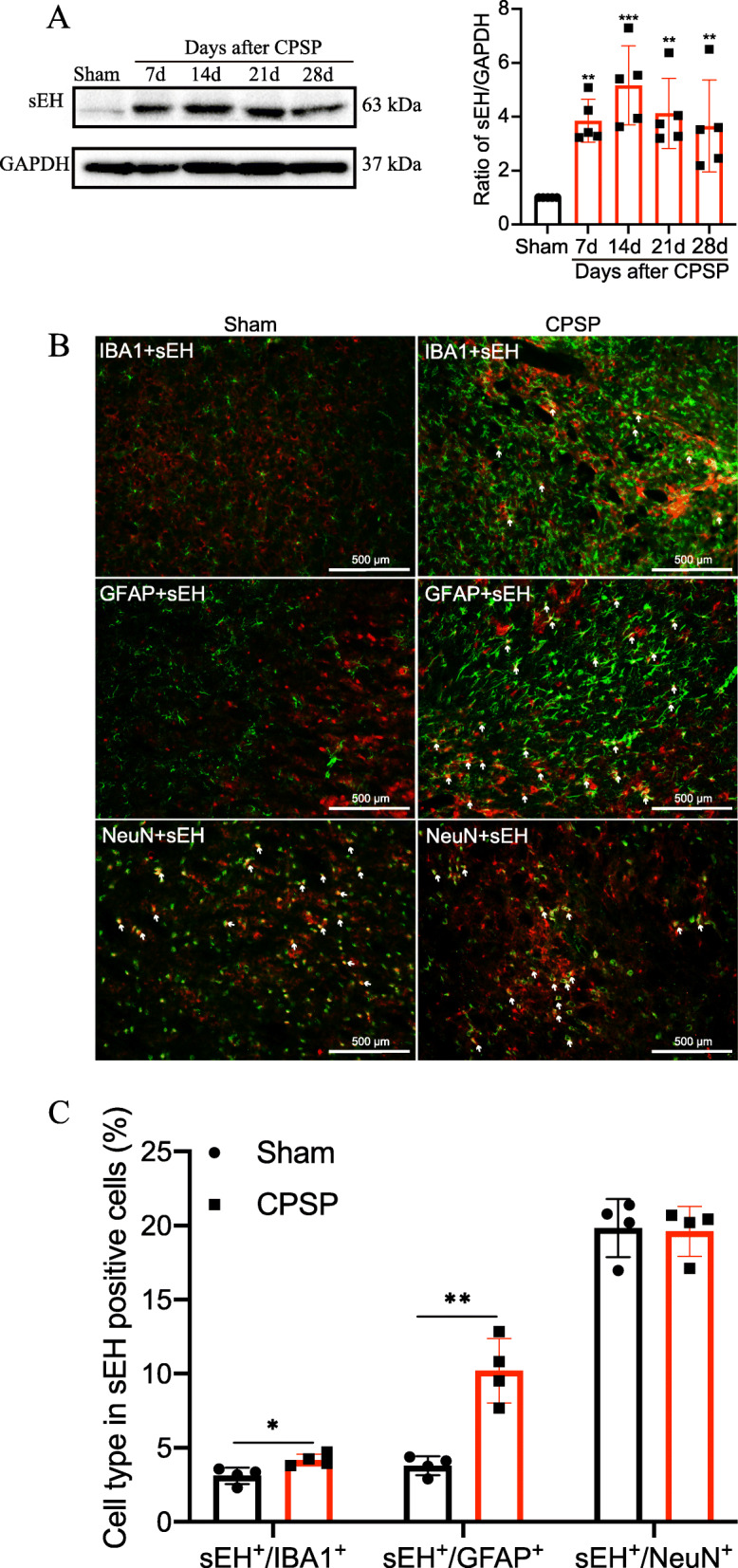
Changes of sEH expression in the perithalamic lesion site of CPSP rats. **a** Representative Western blot bands were presented on the left, with data analysis shown on the right revealing that sEH protein expression is increased around CPSP rat thalamic lesion site, beginning at postlesion day 7 and at least lasting to day 28 after lesion, compared with the sham control. Values are expressed as mean ± SD. GAPDH serves as loading control. **P < 0.01, ***P < 0.001, *n* = 5 rats per group, one-way ANOVA followed by Bonferroni post hoc test. **b** The tissue sections from the injured side thalamus are double immunostained with sEH (red) and reactive astrocyte marker GFAP (green), microglial marker IBA1 (green), or neuron-specific nuclei marker NeuN (green). **c** The percentage of different cell types in sEH-positive cells on day 14 after CPSP induction. *n* = 4 rats per group, *P < 0.05, **P < 0.01, compared with the sham group. Scale bars = 500 μm. IBA1, ionized calcium-binding adapter molecule 1; NeuN, neuronal nuclei; GFAP, glial fibrillary acidic protein

Our previous work revealed that a substantial reduction in 14,15-EET was involved in the development of mechanical allodynia in CPSP rats [24]. We wondered if this was attributed to the change in its hydrolase enzyme sEH in this painful condition. Here, we found that the expression of sEH was markedly increased at 7, 14, 21, and 28 days after hemorrhagic stroke and reached its highest value at day 14 compared with the sham control (Fig. 2a), which is consistent with the time point when rats developed mechanical allodynia and exhibited a slump of 14,15-EET [24]. Moreover, we performed double immunofluorescence staining to investigate the cellular localization of sEH in the thalamus under sham and CPSP conditions. We used cell-specific markers, including IBA-1 for microglia, GFAP for astrocytes, and NeuN for neurons (Fig. 2 b and c). We found that sEH was mainly localized in the neurons of the thalamus of the sham control rats. However, a significant increase in the immunoreactivity of sEH and GFAP was observed in the perithalamic lesion site in CPSP rats. While sEH partially overlapped with IBA-1, its colocalization with neurons around the lesion site remained at the basal level in CPSP rats. This result indicated that the overproduction of sEH along the lesion site under CPSP conditions mainly arises from activated glial cells, especially astrocytes.

### Activation of ER stress and MAPK signaling pathway in the perithalamic lesion site of CPSP rats

**Fig. 3:**
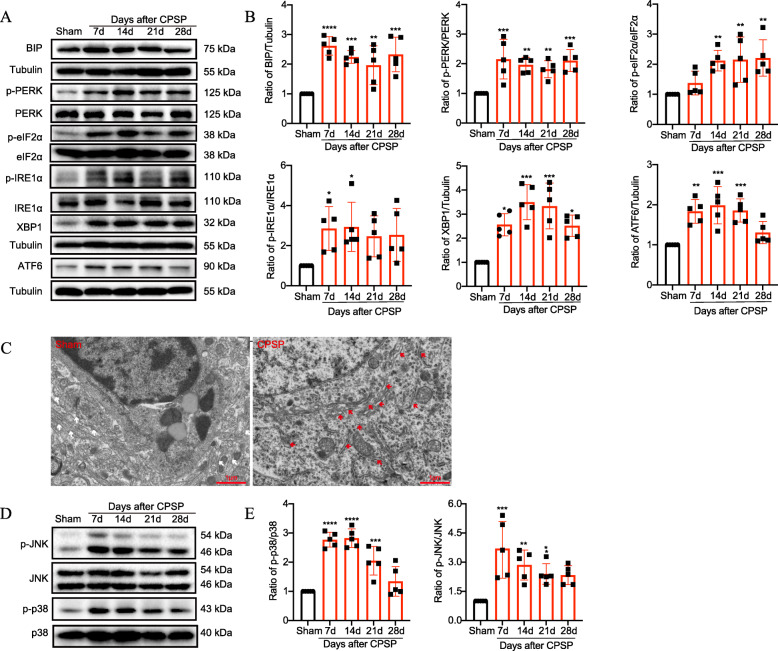
Activation of ER stress and the MAPK-mediated inflammatory pathway in the perithalamic lesion site of CPSP rats. **a**, **b** Representative Western blot bands and quantification of ER stress markers in the perilesion site of sham and CPSP group were presented. The abundance of ER stress markers, including BIP, p-IRE1α, p-PERK, and ATF6 and their downstream targets p-eIF2α and sXbp1, are significantly elevated along the thalamic lesion site during the 1-month observation period after CPSP compared with the sham control. Values are expressed as mean ± SD. The expression of ER stress markers in the sham group were set as 1 for quantification purpose. A scatter plot with a bar chart displays the target expression normalized to Tublin. The levels of phosphorylation are normalized to the total protein. *P < 0.05, **P < 0.01, ***P < 0.001, ****P < 0.0001, *n* = 5 rats per group, one-way ANOVA followed by Bonferroni post hoc test. **c** Electron microscopic observation of the subcellular morphological change of the neurons around the lesion site on day 14 after CPSP induction. The white rows indicate the normal ER, whereas the red arrows show the swollen ER after lesion. Scale bars = 1 μm. **d**, **e** Representative Western blot bands and quantification of JNK and p38 in the perilesion site of sham and CPSP group were presented. The expression of JNK and p38 in the sham group were set as 1 for quantification purposes. A scatter plot with a bar chart displays the target expression normalized to Tublin. The levels of phosphorylation are normalized to the total protein. Phosphorylation of JNK and p38 are significantly increased along the thalamic lesion site, indicating that the MAPK-associated inflammatory pathway is activated after CPSP. Values are expressed as mean ± SD. *P < 0.05, **P < 0.01, ***P < 0.001, ***P < 0.0001, compared with sham control, *n* = 5 per group, one-way ANOVA with the Bonferroni post hoc test

Given that ER stress plays a vital role in the peripheral nervous system alterations in both acute inflammatory pain and chronic neuropathic pain, we examined whether ER stress was also activated upon central nervous system (CNS) injury, particularly after thalamic hemorrhage, and if it contributed to central pain. Compared with the sham control, CPSP rats exhibited a significant increase in levels of ER stress markers, including BIP, p-IRE1α, p-PERK, and ATF6 (Fig. 3 a and b) along the lesion site. In addition, the expression of their downstream targets (p-eIF2α and spliced X-box-binding protein 1 [sXbp1]) in the perilesional tissue were upregulated, suggesting full-scale activation of the subsequent UPR pathways. Moreover, morphological studies using electron microscopy (EM) revealed swollen ER lumens (red arrows shown on the right side of Fig. 3c) throughout the majority of neurons along the lesion site on day 14 after CPSP.

A dysregulated response to ER stress can lead to organ-specific inflammation. For example, the transcription factor XBP1 was identified as a risk factor for human inflammatory bowel disease [29, 30]. Thus, we next focused on the interactions between ER stress and inflammation in our CPSP model. Findings in intestinal epithelial cell lines and brain dopaminergic neurons stated that XBP1-mediated inflammation arose from increased JNK activity, which led us to test whether ER stress, through such actions on the JNK-MAPK pathway, causes excessive neuroinflammation in thalamic tissue after CPSP [29, 31]. Accordingly, we examined MAPK signaling hallmarks by Western blot analysis. As shown in Fig. 3 d and e, compared with the sham control, phosphorylated p38 and JNK were significantly increased in the perilesional thalamic tissue after CPSP, indicating activation of MAPK signaling.

### ER Stress and neuroinflammation is mutually promotive in pain development

**Fig. 4:**
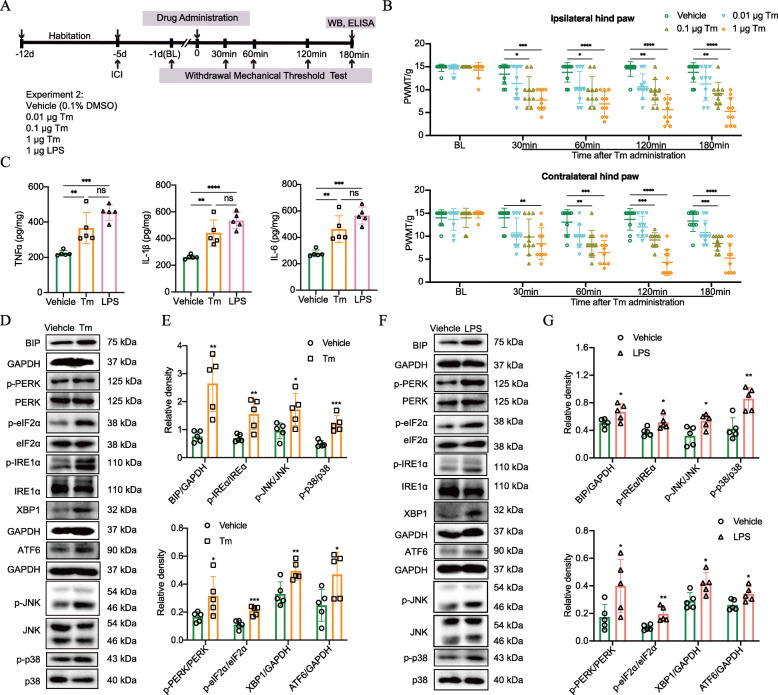
Intrathalamic application of Tm induces mechanical allodynia and neuroinflammation in healthy rats, and vice versa. LPS activates ER stress in healthy rats. **a** The experimental timeline of intrathalamic cannula implantation, agent delivery, pain behavioral tests, and Western blotting. **b** Healthy rats exhibit a significant decrease in PWMT in both ipsilateral and contralateral hind paws throughout the 180-min observation time after Tm administration at a dose of 0.1 μg or 1 μg. *P < 0.05, **P < 0.01, ***P < 0.001, ****P < 0.0001 compared with vehicle-treated rats, *n* = 10 per group, two-way ANOVA with Bonferroni tests. **c** Healthy rats exhibited increased secretion of the proinflammatory cytokines including TNFα, IL-1β, and IL-6 around thalamic VPL at 180 min after intrathalamic injection of 1 μg Tm or 1 μg LPS, compared with the vehicle. Data are expressed as mean ± SD and analyzed by one-way ANOVA with the Bonferroni test. **P < 0.01, ***P < 0.001, ****P < 0.0001 compared with the vehicle-treated rats, *n* = 5 per group. **d**, **e** Representative Western blot bands and quantification of ER stress markers and JNK/p38 in the perithalamic site of vehicle and Tm group were presented. Phosphorylation of JNK and p38, as well as ER stress markers, are significantly elevated around the thalamic VPL region at 180 min after Tm injection in the healthy rats compared with rats given vehicle treatment. Scatter plots with bar graphs display the relative density of the target proteins. Data are expressed as mean ± SD and analyzed by independent t-test. *P < 0.05, **P < 0.01, ***P < 0.001compared with the vehicle-treated rats, *n* = 5 per group. **f**, **g** Representative Western blot bands and quantification of ER stress markers and JNK/p38 in the perithalamic site of vehicle and LPS group were presented. ER stress markers, as well as hallmarks of the MAPK pathway, are markedly increased around the thalamic VPL region at 180 min after LPS injection in the healthy rats. Data are expressed as mean ± SD and analyzed by independent t-test. *P < 0.05, **P < 0.01, compared with the vehicle-treated rats, *n* = 5 per group. Tm, tunicamycin; LPS, lipopolysaccharide; ICI, intrathalamic cannula implantation; ns, no significance

To test whether ER stress that occurred around the lesion site contributed, at least in part, to the pain behavior of CPSP rats, we applied the ER stress inducer Tm to the corresponding thalamic VPL region in healthy rat brains and measured their mechanical pain sensitivity. The timeline of this test (experiment 2) is displayed in Fig. 4a. After 7-day acclimation, the healthy experimental rats were implanted with infusion guide cannulas above the VPL region, as described previously. PWMT was assessed using the von Frey test 1 day before (baseline, BL), and 30, 60, 120, and 180 min after Tm administration with three predetermined doses of 0.01, 0.1, and 1 μg. After that, animals were sacrificed humanely for Western blot and ELISA analysis. We found that rats that received moderate (0.1 μg) and high (1 μg) doses of Tm exhibited significantly decreased response thresholds to mechanical stimulation compared with the mice given vehicle treatment (0.1% DMSO) (Fig. 4b). Meanwhile, the proinflammatory cytokines including TNFα, IL-1β, and IL-6 were significantly increased around thalamic-VPL tissue at 180 min after Tm (1 μg) injection, compared with the vehicle (Fig. 4c). Notably, phosphorylated JNK and p38, as well as ER stress markers, were significantly elevated in response to Tm (1 μg) treatment (Fig. 4 d and e), indicating that ER stress could independently activate inflammation, particularly the MAPK-mediated inflammatory pathway. To test whether neuroinflammation, in turn, exacerbates ER stress in the CNS, we intrathalamically injected 1 μg LPS, the dose of which was previously shown to induce neuroinflammation in the rat brain [32], and then observed for any changes in ER stress hallmarks. We found that along with the expected enhanced secretion of proinflammatory cytokines, increased expression of BIP, p-IRE1α/XBP1, p-PERK/p-eIF2α, and ATF6 were detected in LPS-treated rats, compared with the vehicle, as revealed by Western blot and ELISA analysis (Fig. 4 c, f, and g). Taken together, these results suggest that ER stress is closely interdependent and mutually promotive with neuroinflammation during brain injury, with their interactions becoming a vicious cycle that facilitates nociception in CPSP rats.

### Inhibition of sEH helped alleviate CPSP-elicited mechanical allodynia in a dose- and time-dependent manner

**Fig. 5:**
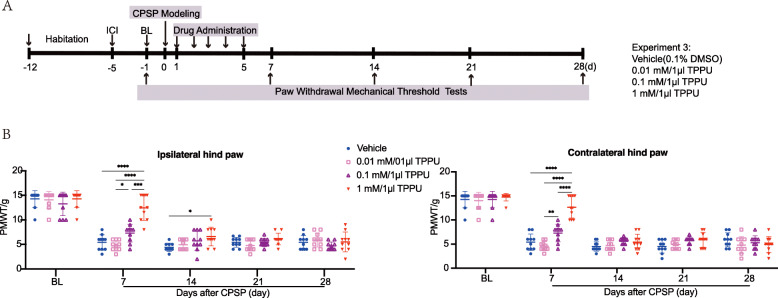
sEHi TPPU alleviates CPSP-induced mechanical allodynia in a dose and time-dependent manner. **a** The time schedule of the present experiment. After 7-day acclimation, rats were subjected to thalamic hemorrhage and were then treated with vehicle (0.1% DMSO) or a different dose of TPPU (0.01 mM, 0.1 mM, 1 mM) once daily within 5 consecutive days after stroke. Rats in each treatment were analyzed by von Frey tests for PWMT at 1 day before (baseline, BL), and 7, 14, 21, and 28 days post stroke. **b** TPPU reduces pain in a dose and time-dependent manner. On day 7 after CPSP, rats treated with a moderate (0.1 mM) or high dose of TPPU (1 mM) exhibited increased PWMT in both hind paws compared to those treated with the vehicle or a relatively low dose of TPPU (0.01 mM). However, this analgesic effect did not last long, with PWMT almost returning to baseline within 9 days after drug withdrawal (at day 14 after CPSP). Data are expressed as mean ± SD, *n* = 10 per group, two-way ANOVA, followed by Bonferroni tests, *P < 0.05, **P < 0.01, ****P < 0.0001

As data from both the in vivo CPSP model employed here and our previous results suggest that sEH-mediated EET degradation plays a key role in nociception, we investigated this further by pharmacological interference with the sEH using inhibitor TPPU and then measured its impact on mechanical allodynia after CPSP. The timeline of this test (experiment 3) is displayed in Fig. 5a. CPSP rats were randomly divided into four groups, with each receiving different doses of TPPU (0.01, 0.1 mM, and 1 mM) or vehicle (0.1% DMSO) once daily via an intrathalamic cannula within the first 5 days after lesion. The von Frey test was performed 7, 14, 21, and 28 days after CPSP induction. We found that a low dose of TPPU (0.01 mM) had no effect on the PWMT of CPSP rats, whereas either a moderate (0.1 mM) or high (1 mM) dose significantly increased the PWMT of both hind paws on day 7 post medication. A high dose notably produced over a 3-fold increase in PWMT compared with the vehicle, thus showing the highest analgesic potency. However, this effect did not last long, with a sudden decrease (based on the value with less or no significance) seen on day 7 of the observation month. These results indicated that TPPU reduced CPSP in a dose- and time-dependent manner (Fig. 5b).

### Inhibition of sEH alleviated er stress and neuroinflammation along the perithalamic lesion site in CPSP rats

**Fig. 6:**
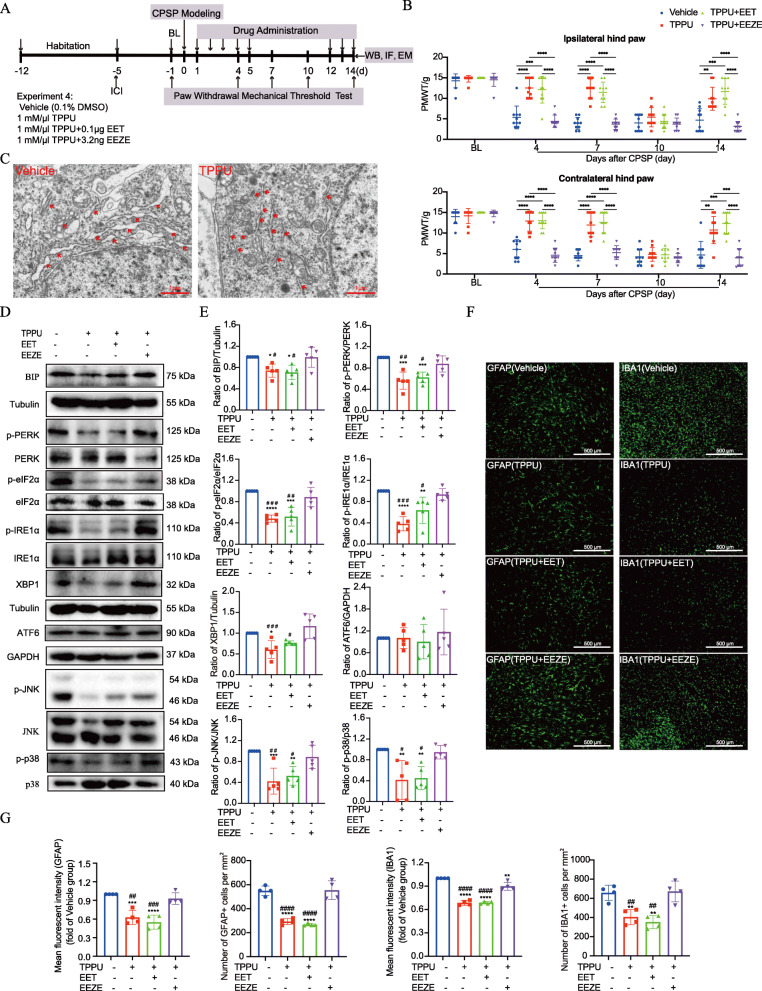
TPPU attenuates mechanical allodynia, ER stress, and neuroinflammation by EET signaling in CPSP rats. **a** The time schedule of the present experiment. After 7-day acclimation, rats were subjected to thalamic hemorrhage and were then treated with the vehicle (0.1% DMSO) or TPPU (1 mM) alone, or in combination with exogenous 14,15-EET (0.1 μg) or 14,15-EEZE (3.2 ng) once daily within the first 5 days after stroke. This was followed by a 6-day treatment-free interval and repeat doses at days 12, 13, and 14. Rats in each treatment group were analyzed by von Frey tests for PWMT at 1 day before (baseline, BL) and at 4, 7, 10, and 14 days post stroke. Besides, Western blot (WB), immunofluorescent staining (IF), and electron microscopic (EM) detection were performed on day 14 after lesion to measure the effects of TPPU/EETs on ER stress and neuroinflammation. **b** Both TPPU and TPPU+14,15-EET-treated CPSP rats exhibited increased PWMT in both hind paws on day 4 during the first drug delivery phase, with this analgesic activity still effective on day 7 during the treatment-free interval until day 10, the effect of which completely disappeared given that the PWMT was longer different from the vehicle-treated group. However, the repeat doses on days 12, 13, and 14 regained their efficacy on allodynia on day 14. These ameliorations were completely blocked by 14,15-EEZE application. Data are expressed as mean ± SD, *n* = 10 per group, two-way ANOVA, followed by Bonferroni tests, **P < 0.01, ***P < 0.001, ****P < 0.0001. **c** EM observation of the subcellular morphological change of the neurons around the lesion site on day 14 after CPSP. The red rows of the left panel indicate the ribosomes associated with highly dilated ER membranes in the vehicle-treated CPSP rats, whereas this dilation was alleviated by TPPU administration (as shown by the red arrows of the right panel). Scale bars = 1 μm. **d**, **e** Representative Western blot bands and quantification of ER stress markers and JNK/p38 in the perilesion site of vehicle, TPPU, TPPU + EET, and TPPU + EEZE group were presented. Except for ATF6, the expression of ER stress markers and the phosphorylation of JNK and p38 around the lesion site were significantly decreased in both TPPU and TPPU + 14,15-EET-treated CPSP rats on day 14 after lesion compared with the vehicle-treated rats, shown by Western blot. This effect was completely eliminated by co-application with 14,15-EEZE. *P < 0.05, **P < 0.01, ***P < 0.001, ****P < 0.0001 compared with the vehicle-treated group, ^#^*P* < 0.05, ^##^*P* < 0.01, ^###^*P* < 0.001 compared with the TPPU+EEZE group, *n* = 5 per group, one-way ANOVA with Bonferroni’s post hoc test. **f**, **g** Immunostaining and quantification of GFAP^+^ and IBA1^+^ cells around the injured thalamic-VPL region on day 14 after lesion. *n* = 4 rats per group, **P < 0.01, ***P < 0.001, ****P < 0.0001 compared with the vehicle group, ^##^*P* < 0.01, ^###^*P* < 0.001, ^####^*P* < 0.0001 compared with the TPPU + EEZE group. Scale bar = 500 μm

Since early treatment with TPPU alone showed significant but relatively short-term analgesia within 7 days after thalamic hemorrhage, groups of CPSP rats were treated with a combination of TPPU (1 mM) and exogenous 14,15-EET (0.1 μg) or EET antagonist 14,15-EEZE (3.25 ng) within the first 5 days after lesion, then received repeat doses at days 12, 13, and 14, and were examined with the von Frey test on days 4, 7, 10, and 14. The timeline of this test (experiment 4) is displayed in Fig. 6a. We found that CPSP rats exhibited increased PWMT on days 4 and 7 after early treatment with TPPU alone or in combination with 14,15-EET compared with the vehicle. However, the addition of exogenous 14, 15-EET did not extend the efficacy of TPPU for analgesia. During the 6 days (from day 6 to day 11) dose interval, the analgesic effect of TPPU disappeared as expected, but repeat dose exposure on days 12, 13, and 14 regained its efficacy on analgesia at day 14 (Fig. 6b). These ameliorations were completely blocked by 14,15-EEZE application, thereby indicating that the analgesic effect of TPPU could be attributed to the actions of EETs.

Next, we investigated whether TPPU acts on the expression of ER stress and MAPK signaling under CPSP conditions. Measurements were taken on day 14 after thalamic hemorrhage. Compared with the vehicle-treated CPSP group, administration of TPPU alone or TPPU plus 14,15-EET significantly reduced the protein levels of BIP, p-IRE1α/XBP1, and p-PERK/p-eIF2α, which are the initiator proteins of the first two branches of the UPR in the perithalamic lesion site. However, no significant difference was observed in the third branch marker, ATF6 (Fig. 6 c and d). Notably, besides affecting the molecular signaling of ER stress, TPPU also reduced the ER dilation in neurons around the lesion site, compared with the vehicle-treated CPSP rats (Fig. 6e). Meanwhile, the relative levels of p-p38 and p-JNK were also markedly decreased by treatment of TPPU alone or in combination with 14,15-EET. However, these decreases were completely abolished when 14,15-EEZE was co-applied, as shown by Western blotting (Fig. 6 c and d). In addition, the immunostaining and quantification shown in Fig. 6 f and g revealed that administration of either TPPU or TPPU plus 14,15-EET alleviated CNS glial cell activation following CPSP, the effect of which could also be reversed by EEZE.

### An ER stress inducer abolished TPPU-mediated pain relief in CPSP rats

**Fig. 7:**
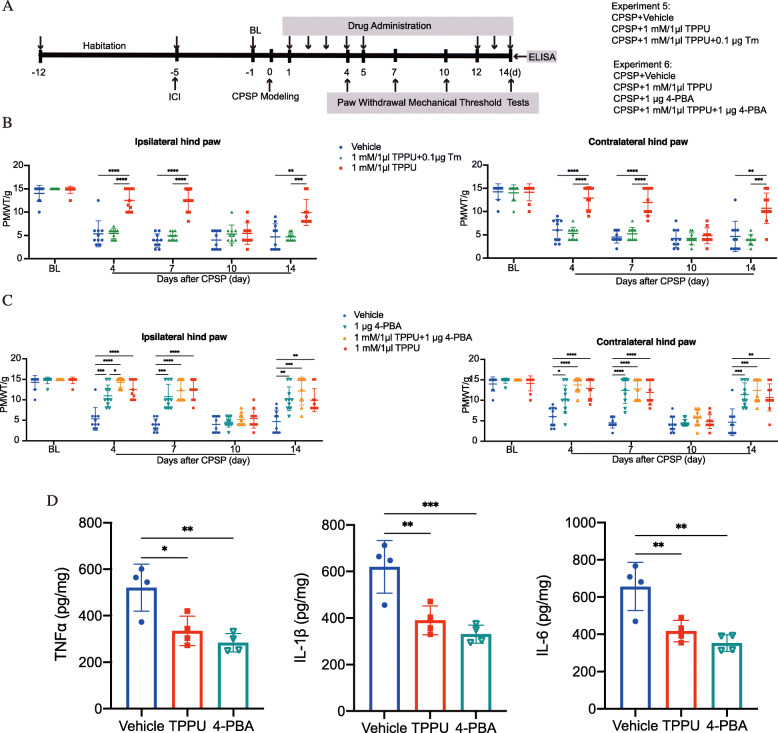
The ER stress inducer Tm abolished the analgesic effect of TPPU in the CPSP rats. **a** The timeline of the present experiment. **b** Application of the ER stress inducer Tm (0.1 μg) completely reversed the analgesic effect of TPPU (1 mM/1 μl) in both hind paws under CPSP conditions during the postlesional 14-day observation period. Data are expressed as mean ± SD. **P < 0.01, ***P < 0.001, ****P < 0.0001, *n* = 10 per group, two-way ANOVA followed by Bonferroni tests. **c** Administration with ER stress inhibitor 4-PBA alone induced a marked suppression of mechanical allodynia in both hind paws in CPSP rats. However, treating CPSP rats with a combination of 4-PBA and TPPU did not result in further attenuation of mechanical allodynia, as no difference was found among the TPPU, 4-PBA, and TPPU + 4-PBA groups. Data are expressed as mean ± SD. *P < 0.05, **P < 0.01, ***P < 0.001, ****P < 0.0001compared with vehicle group, *n* = 10 per group, two-way ANOVA followed by Bonferroni tests. **d** Either TPPU (1 mM/1 μl) or 4-PBA (1 μg) reduced the secretion of proinflammatory cytokines including TNFα, IL-1β, and IL-6 around the perilesion site of the thalamus at day 14 after CPSP induction. Data are expressed as mean ± SD. **P < 0.01, ***P < 0.001, compared with vehicle-treated CPSP group, *n* = 4 per group, one-way ANOVA followed by Bonferroni tests

To further confirm that the analgesic effect of TPPU was, at least in part, due to inhibition of ER stress, we treated CPSP rats with a combination of TPPU and the ER stress inducer Tm or inhibitor 4-PBA and then recorded PWMT on postlesion days at different time points. The schedule for the drug delivery and testing (Experiments 5 and 6) is shown in Fig. 7a. In line with the above experiments, TPPU significantly increased the PWMT of CPSP rats on days 4, 7, and 14. This effect was abolished when 0.1 μg Tm was co-administered (Fig. 7b). This result led us to check whether co-administration of TPPU and 4-PBA (the authentic chemical chaperone aiding in protein folding in the ER) may synergistically produce CPSP relief. Here, we found that administration of 1 μg 4-PBA alone was effective in elevating mechanical nociceptive thresholds. However, combination treatment (TPPU plus 4-PBA) exhibited the same potency as TPPU or 4-PBA alone (Fig. 7c), and meanwhile, for the proinflammatory cytokines release, either TPPU (1 mM/1 μl) or 4-PBA (1 μg) could reduce the levels of TNFα, IL-1β, and IL-6 around the perilesion site of the thalamus at day 14 after CPSP induction, compared with the vehicle-treated CPSP group (Fig. 7d), indicating that TPPU had overlapping functions of being an ER stress inhibitor in suppressing allodynia and the associated neuroinflammation in CPSP rats.

## Discussion

UPR is considered a beneficial physiological response to ER stress, as it orchestrates the transcriptome and proteome in the cell to increase the adaptive capacity of the ER and maintain homeostasis. However, sustained ER stress is believed to overwhelm the protective mechanism of the UPR, leading to unfolded protein accumulation and deposits that promote inflammation, cellular toxicity, and death. Thus, ER stress developed in the CNS has been thought to play fundamental pathogenic roles in many neurodegenerative diseases, such as Parkinson’s disease, Alzheimer’s disease, and amyotrophic lateral sclerosi s[33–35].

In recent decades, an increasing number of studies have indicated that ER stress in the peripheral nervous system, dorsal root ganglion (DRG), and spinal cord contribute to the modulation of nociceptive signal transmission [36–38]. Yamaguchi et al. reported that elevated levels of CHOP, XBP-1, and GRP78 were observed in DRG neurons of spinal nerve ligation (SNL) rats [37]. Moreover, increased expression of IREa, ATF6, and PERK was detected in both sciatic and skin samples in a painful diabetic peripheral neuropathy (DPN) model, while systemic administration of 4-PBA significantly ameliorated both ER stress and mechanical allodynia [38]. In addition, in a rat model of formalin-induced pain, an increased abundance of BIP, ATF6, and p-PERK was detected in the ipsilateral lumbar enlargement of the spinal cord, while pretreatment with 4-PBA before formalin injection significantly reduced pain behaviors in the second (tonic) phase of the pain response [13]. However, beyond the involvement of ER stress in peripheral inflammatory and neuropathic pain, little is known about how ER stress affects the thalamus, the chief center in the CNS for the processing of nociception, and contributes to central pain. In the present study, we observed swollen ER lumens throughout the majority of neurons along the thalamic lesion site, with upregulated expression of the three major ER stress sensors, p-IRE1α, p-PERK, and ATF6, and their downstream targets starting at day 7 after CPSP induction, and lasting for at least 1 month. This result indicated a prolonged full-scale activation of the UPR under thalamic hemorrhage induced-CPSP conditions. In addition, inducing ER stress at the level of the thalamus in healthy rats generated an immediate but relatively lasting painful phenotype. Notably, we also found that administration of 4-PBA alone induced a marked suppression of mechanical allodynia in both hind paws in CPSP rats. Accordingly, a causal association between the ER stress response and CPSP was proposed in this study.

Neuronal injury that causes neuropathic pain not only affects the sensory projection pathways but also leads to a robust immune response at the damaged site. In our study, we found that hemorrhagic injury to the thalamic VPL can cause profound activation of resident immune-like glial cells around the perilesion site for at least 1 month. Activated microglia and astrocytes release a variety of pro-inflammatory mediators, such as IL-1β, IL-6, and TNF-α [39]. The released cytokines and chemokines further activate glial cells and caused hyper-release of mediators from the injury site, which enables a positive feedback loop leading to excessive central neuroinflammation and sensitization.

As both ER stress and neuroinflammation were involved in the CPSP state in this study, we wondered if an interaction between these two pathological pathways exists to conspire to initiate and progress CPSP. Accumulating evidence suggest that cells under severe ER stress could induce a UPR-dependent inflammatory pathway, which further exacerbates the innate inflammation caused by the original insult [40].

The transcription factor XBP1, which is activated by the ER transmembrane protein IRE1, is an important component of UPR. Kaser et al. reported that mice lacking XBP1 in intestinal endothelial cells (IECs) exhibited inefficient UPR, leading to an increase in ER stress, hyperactivation of IRE1, and increased JNK phosphorylation, thus further inducing inflammatory responses in IECs and resulting in the development of spontaneous inflammatory bowel disease (IBD) [29, 30]. Moreover, Wei et al. demonstrated that heart failure rats exhibited increased ER stress and pro-inflammatory cytokine release in their subfornical organ and hypothalamic paraventricular nucleus, and that the application of inhibitors selective for p38 and JNK reduced brain ER stress and inflammation in the cardiovascular regulatory regions of the brain, thus resulting in decreased sympathetic excitation in heart failure rats [41, 42]. In addition, Urano et al. and Shaulian et al. revealed the detailed molecular mechanism of the UPR-MAPK-inflammation pathway, where IRE-1α recruits the adaptor protein TRAF2, which activates apoptosis signal-regulating kinase 1(ASK1), and then coordinates the activation of JNK. IRE-1α-dependent activation of JNK stimulates the basic leucine zipper (bZIP) transcription factor activator protein 1 (AP-1). Thereafter, AP-1, a heterodimer composed of a differential combination of Fos, Jun, ATF, and Maf subfamily members, binds to enhancer elements that upregulate the transcription of inflammatory genes [43, 44]. Taken together, these results indicate that the MAPK family JNK and p38 are the principal inflammatory proteins activated during UPR. Thus, we chose JNK and p38 as our key targets to explore the interaction between ER stress and neuroinflammation under CPSP conditions. Here, we found that, along with the elevated UPR responses, phosphorylated p38 and JNK were significantly increased in the perilesional thalamic tissue after CPSP, indicating activation of MAPK signaling. Next, to test whether ER stress, through such actions on the JNK-MAPK pathway, caused excessive neuroinflammation and pain, we designed an experiment with healthy rats intrathalamically injected with Tm. We found that concurrent with the pain behavior, the MAPK pathway was activated immediately after Tm administration. This rapid effect suggests that phosphorylation of the key targets, p38 and JNK, may be the intrinsic mechanism influenced by ER stress.

While studying EETs, we reported a slump of 14,15-EET in the thalamus of CPSP rats [24]. Here, we found that sEH was elevated along the perilesion site of CPSP brains and was associated with cerebral immuno-responsive glia, especially reactive astrocytes. This observation confirmed previous findings [45], suggesting that sEH-mediated degradation of EETs after hemorrhage may play a vital role in the progression of CPSP. In the present study, inhibition of sEH by TPPU led to alleviation of allodynia and reduction of ER stress response, glia activation, proinflammatory cytokine release, as well as MAPK activation resulting from CPSP. Importantly, we demonstrated that co-administration of TPPU and the standard chemical chaperone 4-PBA did not synergistically block the pain-related behavior in CPSP rats, suggesting that these two agents converged on the same ER stress pathway and reached a ceiling effect in assisting the correct folding of nascent proteins as well as blocking the associated neuroinflammation under CPSP conditions.

**Fig. 8:**
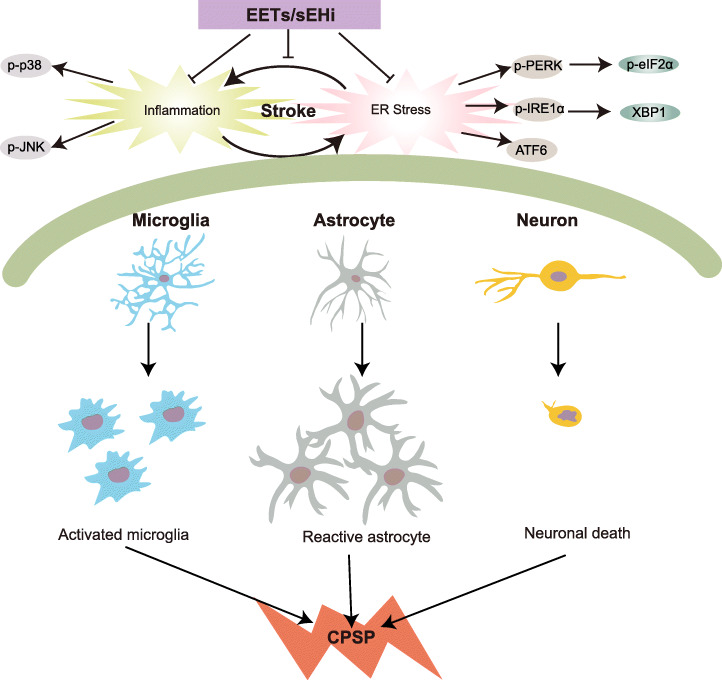
Schematic diagram showing the involvement of EETs/sEHi in modulating the crosslink between ER stress and neuroinflammation under stroke and the subsequent CPSP condition. Stroke activates the ER stress response (UPR) to protect cells against toxic accumulation of misfolded proteins, as well as help maintain the functional integrity of ER. However, prolonged UPR instructs the stressed neurons to commit suicide by triggering apoptosis, which elicits a defensive innate immune response against the invading pathogens that cause glial cell activation and excessive neuroinflammation. The excessive neuroinflammation, in turn, exacerbates ER stress. The vicious interactions between ER stress and neuroinflammation result in central sensitization and pain and deleteriously contribute to stroke damage, the suppression of which by sEHi/EETs may provide a therapeutic approach for stroke and CPSP

Overall, our findings provide insight into the mechanism underlying CPSP, where the fundamental cellular and molecular events are driven by the crosstalk between ER stress and neuroinflammation (Fig. 8). Stabilizing bioactive EETs by sEHI at the initial onset of stroke may block the feed-forward ER stress-neuroinflammation loop. This effect, together with the ability to suppress central disinhibition through neurosteroid-GABA signaling, revealed in our previous study [24], indicates that preserving the levels of endogenous EETs by sEHI has great potential for the control of central sensitization and its associated central neuropathic pain.

A limitation of this study is that we only included male animals, which may not have accounted for sex-dependent differences in pain response. Experimental data revealed that interactions among biological, psychological, and sociocultural factors of men and women probably contribute to these differences. Of particular interest, estrogen, which is essential for the female reproductive system, appears to exert a positive effect on anti-nociception. Lee et al. showed that estrogen alleviated neuropathic pain induced after spinal cord injury by inhibiting microglia and astrocyte activation [46], while Deng et al. found that estrogen reduced mechanical and thermal pain thresholds in rats with sciatic nerve constriction by upregulating spinal NMDA receptor activity in the dorsal root ganglion [47]. To exclude the effect of estrogen on pain, we only included male rats in our experiment. However, sex differences in central neuropathic pain, including pain threshold, drug response, and tolerance, remain critical and largely unanswered questions that should be further studied. Thus, female mice should also be used in future studies of CPSP. It should also be noted that the MAPK pathway not only regulates the inflammatory gene expression but also play a role in mediating cell survival, the mechanisms by which ER and oxidative stress-induced neuronal cell death contributes to the pathogenesis and progression of central neuropathic pain are of interest, and deserve to be further investigated.

## Conclusion

CPSP is one of the most challenging but poorly understood comorbidities of stroke, with its etiology attributed to excessive central neuronal excitability. The present study provides evidence that the interaction between ER stress and neuroinflammation after stroke forms a vicious cycle that undermines the self-repairing system in the CNS, which ultimately generates central sensitization and sustained pain. Agents that target EET signaling exhibit great potential for treating CPSP by suppressing excessive ER stress and neuroinflammatory responses, as well as reserving the normal thalamic inhibition state. Importantly, a number of small molecule sEHIs including TPPU are currently available, the use of which has been proven to have good blood-brain permeability when applied systemically and is effective in animal models of diverse neuropathic pain without leading to apparent toxicity. Thus, early treatment of sEHIs at the initial onset of stroke has emerged as a promising therapeutic approach for CPSP.

## Data Availability

The data and materials supporting the results in this article are available from the corresponding author on reasonable request.

## Abbreviations

4-PBA: Sodium 4-phenylbutyrate
AP-1: Transcription factor activator protein 1
ARA: Arachidonic acid
ATF6: Activating transcription factor 6
BIP: Binding immunoglobulin protein
bZIP: Basic leucine zipper
CHOP: C/EBP homologous protein
CNS: Central nervous system
Contra: Contralateral
CPSP: Central post-stroke pain
CYP: Cytochrome P450
DHETs: Dihydro-eicosatrienoic acids
EETs: Epoxyeicosatrienoic acid
eIF2α: Eukaryotic initiation factor 2α
ER: Endoplasmic reticulum
GABA_B_R: Gamma-aminobutyric acid B receptors
GFAP: Glial fibrillary acidic protein
HRP: Horseradish peroxidase
IBA1: Ionized calcium-binding adapter molecule 1
IF: Immunofluorescence
IL-1β: interleukin-1β
IL-6: Interleukin-6
LPS: Lipopolysaccharide
Ipsi: Ipsilateral
IRE1α: Inositol-requiring kinase 1α
MPTP: 1-Methyl-4-phenyl-1,2,3,6-tetrahydropyridine
NeuN: Neuronal nuclei
PERK: Protein kinase RNA-like endoplasmic reticulum kinase
PWMT: Paw withdrawal mechanical threshold
sEH: Soluble epoxy hydrolase
sEHi: Soluble epoxy hydrolase inhibitors
Tm: Tunicamycin
TNF-α: Tumor necrosis factor-α
UPR: Unfolded protein response
VPL: Ventral posterior lateral nucleus
WB: Western blot
